# Factors affecting distal false lumen enlargement after thoracic endovascular aortic repair for type B aortic dissection

**DOI:** 10.1016/j.heliyon.2023.e17248

**Published:** 2023-06-15

**Authors:** Qian-hui Tang, Jing Chen, Zhen Long, Yu-Lin Wang, Xuan-an Su, Jian-ye Qiu, Qiu-ning Lin, Jiang-feng Zhang, Xiao Qin

**Affiliations:** Department of Vascular and Endovascular Surgery, The First Affiliated Hospital of Guangxi Medical University, Nanning, 530021, Guangxi, China

**Keywords:** Type B aortic dissection, False lumen enlargement, Thoracic endovascular aortic repair

## Abstract

**Objective:**

To investigate the factors influencing distal false lumen enlargement after thoracic endovascular aortic repair (TEVAR) for type B aortic dissection.

**Materials and methods:**

Data were collected on patients with type B aortic dissection who underwent TEVAR from January 2008 to August 2022. Patients were divided into a distal aortic segmental enlargement (DSAE) group and a non-DSAE group based on whether the distal false lumen was dilated more than 5 mm on computed tomographic angiography (CTA) images. To analyze the independent influences on distal false lumen dilatation after TEVAR, the variables with a *P* value < 0.05 during univariate analysis were included in the binary logistic regression analysis model.

**Results:**

A total of 335 patients were included in this study, with 85 in the DSAE group and 250 in the non-DSAE group. The mean age was 52.40 ± 11.34 years, 289 (86.27%) were male patients, and the median follow-up time was 6.41 (11.99–29.99) months. There were significant differences in Marfan syndrome, chronic obstructive pulmonary disease (COPD), and follow-up time between the two groups. In terms of morphology, there were statistically significant differences in the number of tears, the size of the primary tear, and the length of dissection between the two groups. Binary logistic regression analysis indicated that Marfan syndrome, COPD, and the primary tear size were associated with distal false lumen dilatation.

**Conclusions:**

Marfan syndrome, COPD, and the primary tear size influence distal aortic segmental enlargement after TEVAR in type B aortic dissection patients.

## 1 Introduction

Aortic dissection is a life-threatening vascular event; its symptoms are often mistaken for angina pectoris or pulmonary embolism, with a misdiagnosis rate of 33.8% [1]. Aortic dissection is a lesion caused by blood entering the vascular wall through intimal tears, which can lead to organ or limb ischemia, or aortic rupture. Aortic dissection can be divided into type A aortic dissection, in which the tear is located in the ascending aorta, and type B aortic dissection, in which the tear is located in the left region of the cephalic trunk [2]. Type B aortic dissection accounts for approximately 37.7% of all dissections [3].

Thoracic endovascular aortic repair (TEVAR) is effective in improving aortic dissection remodeling and promoting false lumen thrombosis [3]. TEVAR has become an effective treatment for type B aortic dissection, especially for complicated type B aortic dissection with mal-perfusion syndrome or rupture [4]. However, the current TEVAR technique is flawed because it focuses on the proximal primary tear. The aortic dissection usually has multiple tears, and it is not appropriate to apply a stent graft to cover all of the tears considering the perfusion of the organ. Although thoracic aortic segments were successfully reconstructed after TEVAR, the prognosis for residual distal entrapment was poor, with 62.7% of distal dissection dilating within 5 years [5]. This study was conducted to identify the influencing factors for distal false lumen enlargement. We hypothesize that the morphological features of type B aortic dissection are influential factors in the enlargement of the distal aortic segment after TEVAR.

## 2 Materials and Methods

### 2.1 Study population

Patients diagnosed with type B aortic dissection via computed tomographic angiography (CTA) from January 2008 to August 2022 at the First Affiliated Hospital of Guangxi Medical University were selected. Their demographic information, comorbidities, and procedural details were obtained from the hospital's electronic medical record system. CTA images were collected from the electronic imaging system. Patients were divided into a distal aortic segmental enlargement (DSAE) group and a non-DSAE group based on the presence or absence of a dilated false lumen. Inclusion criteria consisted of: 1) age ≥18 years, 2) undergoing TEVAR treatment, 3) residual distal dissection after TEVAR, and 4) having at least one post-operative CTA review. Exclusion criteria included unavailable CTA images and not undergoing TEVAR at the hospital selected for this study.

All of the procedures were performed based on the ethical standards of the First Affiliated Hospital of Guangxi Medical University Ethics Committee and the Declaration of Helsinki (as revised in 2013). Individual consent for this retrospective analysis was waived since the manuscript contains no identifiable patient information.

### 2.2 Surgical technique and follow-up

If the distance of the primary tear from the origin of the left subclavian artery was >1.5 cm, a conventional TEVAR procedure was performed. If the distance of the primary tear from the origin of the left subclavian artery was not sufficient to be used as an anchorage area for the stent graft, the stent graft covered the origin of the left subclavian artery. The decision to reconstruct the left subclavian artery was based on the condition of the left vertebral artery. Reconstruction of the left subclavian artery was performed using the chimney technique, fenestration technique, or branched stent graft. The chimney technique refers to the insertion of a stent into the branch artery during TEVAR, thereby preserving the branch vessels covered by the aortic stent. The hybrid operation was also implemented where appropriate. All of the patients were asked to return for CTA at 2 weeks, 1 month, 3 months, 6 months, 1 year, 3 years, and 5 years, post-operatively.

### 2.3 Definitions

**Fig. 1:**
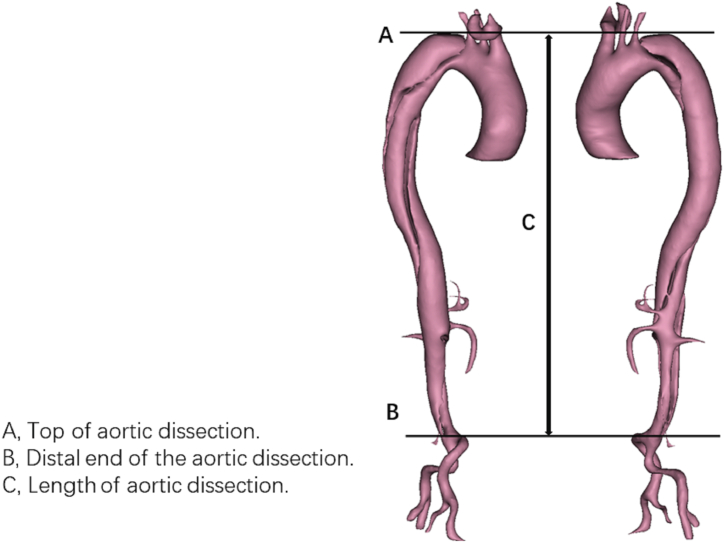
Measurement of the length of aortic dissection.

The aortic dissection tear was identifiable on the cross-section of the CTA image. The length of the aortic dissection was the distance in the vertical direction from the top to the distal end of the aortic dissection (Fig. 1). The primary tear size was the maximum width of the primary tear in the cross-section. Distal aortic segmental enlargement was defined as the enlargement of the post-operative false lumen by more than 5 mm in the cross-section compared to the pre-operative false lumen. All of the measurements were based on CTA images.

### 2.4 Statistical analysis

Continuous data were expressed as means and standard deviations or medians and quartiles based on whether they conformed to a normal distribution. Categorical data were expressed as frequencies and percentages. Continuous data were compared using Student's *t*-test or Mann–Whitney *U* test. Categorical data were compared using the chi-squared test or Fisher's exact test. Variables with *P* values < 0.05 in the univariate analysis were included in a binary logistic regression model to explore independent factors for distal aortic segmental enlargement. All of the statistical analyses were implemented in SPSS software (version 25.0). *P* values < 0.05 were considered to be statistically significant.

## 3 Results

**Fig. 2:**
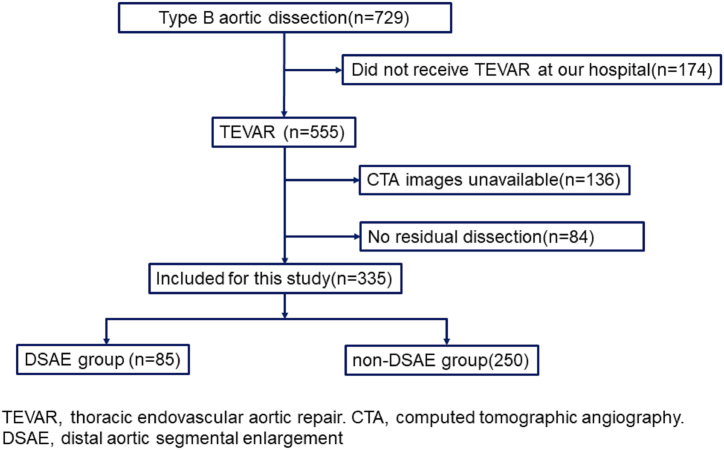
Flowchart of patient selection.

The patient inclusion process is provided in Fig. 2. A total of 729 patients were diagnosed with type B aortic dissection between January 2008 and August 2022, of which 555 patients underwent TEVAR at the First Affiliated Hospital of Guangxi Medical University. A total of 136 patients had no post-operative CTA and were excluded; the final study included 335 patients. The mean age was 52.40 ± 11.34 years, 289 (86.27%) were male patients, and the median follow-up time was 6.41 (1.99–29.99) months. There were 85 patients in the DSAE group and 250 patients in the non-DSAE group. The distribution of Marfan syndrome and chronic obstructive pulmonary disease (COPD) was statistically different between the two groups (*P* < 0.05). In terms of morphology, the number of tears, the primary tear size, the vertical length of aortic dissection, and follow-up time were statistically different between the two groups. The demographic and surgical information of the patients are presented in Table 1, and the morphological information of the aortic dissection is displayed in Table 2.

**Table 1:** Demographics, comorbidities and procedural details features.

Characteristics	DSAE group (85)	non-DSAE group (250）	Total（335）	*P*
Male	70 (82.35)	219 (87.60)	289 (86.27)	0.225
Age (year)	51.24 ± 10.11	52.8 ± 11.72	52.40 ± 11.34	0.272
Weight (kg)	71.25 ± 12.24	70.72 ± 13.16	70.85 ± 12.93	0.761
Height (cm)	167.45 ± 7.01	166.64 ± 6.95	166.84 ± 6.963	0.389
Systolic blood pressure (mmHg)^**a**^	143.89 ± 28.45	150.16 ± 29.55	148.57 ± 29.357	0.089
Diastolic blood pressure (mmHg)^**b**^	84.36 ± 17.53	87.64 ± 16.45	86.81 ± 16.767	0.119
Intensive care unit stays (hours)	24.00 (4.50,24.00)	24.00 (0,24.00)	24.00（0，24.00）	0.856
Follow-up period (months)	15.33 (3.98,50.61)	4.39 (1.72,23.49)	6.41 (1.99,29.99)	0.000
**Symptoms**				
Chest pain	44 (51.76)	141 (56.40)	185 (55.22)	0.458
Chest tightness	17 (20.00)	33 (13.20)	50 (14.93)	0.129
Abdominal pain	23 (27.06)	67 (26.80)	90 (26.87)	0.963
Back pain	28 (32.94)	77 (30.80)	105 (31.34)	0.731
**Comorbidities**				
Coronary artery disease	8 (9.41)	17 (6.80)	25 (7.46)	0.429
Stroke	11 (12.94)	32 (12.80)	43 (12.84)	0.973
Renal insufficient ^**c**^	9 (10.59)	18 (7.20)	27 (8.06)	0.322
Hypertension	67 (78.82)	207 (82.80)	274 (81.79)	0.412
Diabetes	3 (3.53)	21 (8.40)	24 (7.16)	2.263
Marfan syndrome	3 (3.53)	1 (0.40)	4 (1.19)	0.022
Autoimmune disease	6 (7.06)	10 (4.00)	16 (4.78)	0.253
COPD	9 (10.59)	6 (2.40)	15 (4.48)	0.002
Peripheral artery disease	3 (3.53)	3 (1.20)	6 (1.79)	0.162
History of other surgery	9 (10.59)	24 (9.60)	33 (9.85)	0.792
Smoking	34 (40.00)	124 (49.60)	158 (47.16)	0.126
Alcohol intake	38 (44.71）	109 (43.60)	147 (43.88)	0.859
**Procedural details**				
Operation time (min)	149.72 ± 102.15	136.96 ± 102.84	140.19 ± 102.663	0.323
Length of stent graft coverage (mm)	186.4 ± 41.57	187.06 ± 45.24	186.89 ± 44.279	0.118
Hybrid operation	5 (5.88)	22 (8.80)	27 (8.06)	0.393
Partial or total coverage of LSA	25 (29.41)	74 (29.60)	99 (29.55)	0.974
Reconstruction of LSA	13 (15.29)	25 (10.00)	38 (11.34）	0.184

COPD, chronic obstructive pulmonary disease. LSA, left subclavian artery.

a, defined as systolic blood pressure at admission. b, defined as diastolic blood pressure at admission. c, defined as estimated glomerular filtration rate less than 60 mL/min/1.73 m^2^.

**Table 2:** Morphological characteristics of type B aortic dissection.

Characteristics	DSAE group (85)	non-DSAE group (250）	Total（335）	*P*
Number of intimal tears	4.59 ± 2.18	3.94 ± 1.96	4.10 ± 2.036	0.011
Primary tear size (mm)	9.44 ± 5.72	12.13 ± 7.60	11.4437 ± 7.26	0.001
Distance from primary tear to LSA (mm)	19.63 (10.00,30.19)	20.00 (9.05,34.18)	20.00 (9.16,32.38)	0.383
Length of dissection (mm)	376.17 ± 81.16	348.81 ± 98.38	355.75 ± 94.94	0.012
Operation time (min)	149.72 ± 102.15	136.96 ± 102.84	140.19 ± 102.66	0.323
Complicated TBAD	13 (15.29)	46 (18.40)	59 (17.61)	0.516
Preoperative partially thrombosed false lumen	44 (51.76)	120 (48.00)	164 (48.96)	0.549

LSA, left subclavian artery. TBAD, type B aortic dissection.

Marfan syndrome, COPD, the number of tears, the primary tear size, the vertical length of aortic dissection, and follow-up time were included in the binary logistic regression analysis. Correlations were seen between Marfan syndrome, COPD, primary tear size, follow-up time, and DSAE. The results of the binary logistic regression analysis are shown in Table 3.

**Table 3:** Results of binary logistic regression analysis. COPD, chronic obstructive pulmonary disease.

Variable	B	Wald	*p*	OR (95%CI)
COPD	−1.637	7.841	0.005	0.195 (0.062–0.612)
Marfan syndrome	−2.725	4.715	0.030	0.066 (0.006–0.767)
Number of intimal tears	0.111	2.214	0.137	1.118 (0.965–1.294)
Primary tear size (mm)	−0.073	11.110	0.001	0.930 (0.891–0.970)
Length of dissection (mm)	0.003	2.372	0.124	1.003 (0.999–1.006)
Follow-up period (months)	0.021	21.768	0.000	1.021 (1.012–1.030)

## 4 Discussion

TEVAR has become an effective treatment for type B aortic dissection. The 5-year and 10-year survival rates for type B aortic dissection treated with TEVAR have been reported to be 96.5% and 83.0%, respectively [6]. While current TEVAR procedures focus on the primary tear in the thoracic aortic segment, some type B aortic dissection patients still have residual distal dissection after TEVAR. Aneurysmal dilatation of the dissection has become the most common complication of type B aortic dissection in the chronic phase [4]. In contrast, the size and growth rate of aortic dissection have become indicators for re-intervention in chronic stage aortic dissection and distal aortic dissection. The current literature [4,7] states that the persistence of a false lumen, aortic dissection growth rate >10 mm/year, and aortic diameter >55–60 mm can be an indication for surgical treatment of aortic dissection in the chronic phase. Therefore, identifying the factors influencing distal aortic dissection dilatation is beneficial in improving the long-term prognosis of type B aortic dissection.

In this study, the incidence of dilatation was 25.37%, whereas Zhang et al. [8] found that the incidence of dilatation was 13.41%. The source of this discrepancy may stem from the difference in definition, as Zhang et al. [8] defined dilatation as a diameter >1.5 times the normal aortic diameter or a growth rate >10 mm/year. In contrast, the definition of dilatation in this study is consistent with Gasparetto's [9] definition as a 5 mm increase in false lumen diameter compared to the pre-operative period. Gasparetto et al. reported a 19.35% incidence of aortic dissection dilatation in type B aortic dissection patients undergoing TEVAR [9].

Our results suggested that COPD was a risk factor for residual aortic dissection dilatation after TEVAR for type B aortic dissection. This result was consistent with previous reports in the literature [10], and Fujikura et al. also suggested that the more severe the COPD, the larger the diameter of the aorta. The presence of COPD was also a risk factor for the rapid growth of thoracic aortic aneurysms [11]. Another important finding of this study was that there was a statistically significant difference in follow-up time between the DSAE and non-DSAE groups. Based on this result, we hypothesized that there was a tendency for residual aortic dissection to expand and that more dilated lesions could be detected with increased follow-up time. Close follow-up is beneficial for the timely detection of dilated lesions. This hypothesis was supported by the study of Sueyoshi et al. [12], which indicated that aortic dissection in the thoracic and abdominal aortic segments grew at a rate of 4.1 mm/year and 1.2 mm/year, respectively, and that 83.9% of type B aortic dissection patients had dilated aortic dissection during follow-up.

We hypothesized that morphological features of type B aortic dissection were associated with distal aortic segmental enlargement after TEVAR. Zhang et al. [8] also observed that morphological features were associated with distal aortic segmental enlargement after TEVAR, indicating that the location and size of residual tears were major risk factors for patients with DSAE. However, they did not focus on the role of primary tear size in distal aortic segmental enlargement after TEVAR. In our study, primary tear size was associated with distal segmental aortic dilatation after TEVAR, and the smaller the primary tear size, the greater the risk of distal aortic segmental enlargement after TEVAR. It has been suggested that a smaller primary tear size was associated with higher false lumen diastolic pressure [13]. Therefore, we hypothesized that a smaller primary tear size increased the false lumen diastolic pressure and led to distal aortic segmental enlargement after TEVAR.

TEVAR generates positive remodeling of the false lumen by covering the primary tear of the aortic dissection with a stent graft. This reduces the flow velocity and pressure within the false lumen and induces thrombosis. However, if there are multiple tears in the aortic intima, TEVAR only results in converting a large dissection into a small one with blood still flowing into and out of the distal aortic dissection. The prolonged presence of blood flow in the false lumen is a significant risk factor for increased aortic diameter [12].

Hemodynamics is a key factor in false lumen outcomes. Through computational hemodynamic simulation studies, Xu et al. [14] found that the particle relative residence time increased in the stable group and decreased in the enlarged false lumen group. Additionally, Shang et al. [15] revealed that the percentage of total flow passed through the false lumen and the time-averaged wall shear stress on the aortic wall were higher in the rapid aneurysmal degeneration group than in the stable aortic diameter group. Li et al. [16] discovered that, as the number and area of tears increased, the area with an oscillatory shear index >0.45 and the area with an endothelial cell activation potential >1.5 decreased significantly. However, the blood flow into the false lumen increased significantly, and these changes would be detrimental to thrombosis within the false lumen. Based on these findings, it is important to manage the distal residual tears and reduce blood flow within the false lumen to avoid expansion of the distal residual dissection.

The origin of abdominal vessels and distal tears are located simultaneously in the abdominal aorta, which is a difficult area for the treatment of distal residual aortic dissection. This is because the conventional implantation of a stent graft will block the blood supply to the organs, triggering ischemia. Because endoluminal embolization provides an environment conducive to thrombosis, it can be used, not only as a rescue treatment for acute and chronic dissecting aortic aneurysms, but also as an adjunct to TEVAR [[17], [18], [19]]. However, there is a lack of devices specifically designed for false lumen embolization. Shen et al. [20] demonstrated in animal studies that the spiral stent breach closure device offers a new approach to tears located in the branch region of the abdominal aorta. In addition, Cao et al. [21] reported that the ENDOPATCH™ system could be an effective treatment for residual dissection, allowing precise sealing of the distal tear and effective preservation of visceral perfusion. However, this treatment requires long-term follow-up studies. A detailed assessment of the benefits and risks of closing the distal tear is imperative before treatment. Li et al. [22] reported that the role of hemodynamics, including quantification of blood flow through the tears and changes in pressure between the true and false lumen, could be used to predict the risk of closing the distal re-entry.

### 4.1 Limitations

This was a retrospective study with a limited sample and follow-up time. First, a prospective, large sample is required for a more robust assessment. Second, there may have been confounding factors that were not included in our model. Finally, the morphological measurements in this study were based on CTA images, which may have resulted in measurement bias. The accuracy may be enhanced if combined with intravascular ultrasonography.

## 5 Conclusions

The size of the primary tear is an independent predictor of distal aortic segmental enlargement. Patients with Type B aortic dissection combined with chronic obstructive pulmonary disease or Marfan syndrome are vulnerable to distal aortic segmental enlargement after TEVAR.

## Funding

This study was funded by the 10.13039/501100001809National Nature Science Foundation of China (NO. 82260099).

## Ethical statement

The authors are accountable for all aspects of the work in ensuring that questions related to the accuracy or integrity of any part of the work are appropriately investigated and resolved. All procedures performed in this study involving human participants were by the Declaration of Helsinki (as revised in 2013). This study is a single-center retrospective study. And institutional review board approval was not required to conduct this study. Individual consent for this retrospective analysis was waived.

## Author contribution statement

Qian-hui Tang: Conceived and designed the experiments: Performed the experiments; Analyzed and interpreted the data; Contributed reagents, materials, analysis tools or data; Wrote the paper. Jing Chen: Conceived and designed the experiments: Performed the experiments; Analyzed and interpreted the data; Wrote the paper. Zhen Long: Analyzed and interpreted the data; Wrote the paper. Yu-Lin Wang, Xuan-an Su and Jian-ye Qiu: Contributed reagents, materials, analysis tools or data; Wrote the paper. Qiu-ning Lin: Performed the experiments; Wrote the paper. Jiang-feng Zhang: Performed the experiments; Contributed reagents, materials, analysis tools or data; Wrote the paper. Qin Xiao: Conceived and designed the experiments; Analyzed and interpreted the data; Wrote the paper.

## Data availability statement

Data included in article/supplementary material/referenced in article.

## Additional information

No additional information is available for this paper.

## Declaration of competing interest

The authors declare the following financial interests/personal relationships which may be considered as potential competing interests:Xiao Qin reports financial support was provided by The First Affiliated Hospital of Guangxi Medical University. Xiao Qin reports a relationship with The First Affiliated Hospital of Guangxi Medical University that includes: employment and funding grants. Xiao Qin has patent pending to none. The authors have no conflicts of interest to declare.
